# Frontal Ethmoid Mucocele: An Unusual Postoperative Complication

**DOI:** 10.1002/oto2.26

**Published:** 2023-02-23

**Authors:** Nicholas L. Schenck, Sarah A. Ustrell, Zhiheng Chen

**Affiliations:** ^1^ Cedars Sinai Division of Otolaryngology Cedars Sinai Medical Center Los Angeles California USA; ^2^ Cedars‐Sinai Sinus Center Los Angeles California USA; ^3^ Sol Price School of Public Policy University of Southern California Los Angeles California USA

**Keywords:** complications, frontal mucocele, sinus surgery

## Case

**Figure 1:**
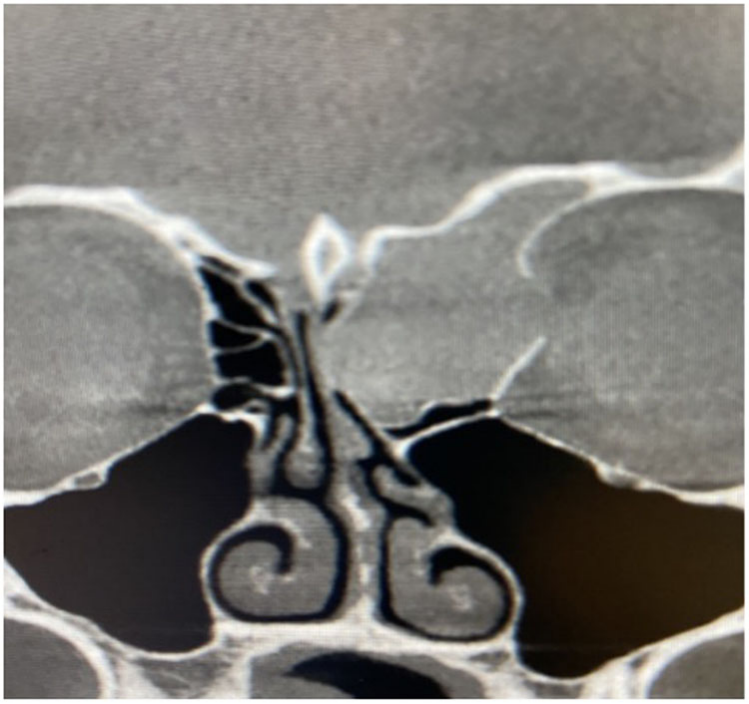
Computed tomography sinus. It shows frontal ethmoid mucocele destroying the left lamina papyracea. Note intact frontal sinus.

A 23‐year‐old female initially presented with a 2‐month history of severe frontal sinus headaches and upward gaze diplopia. She had no other significant medical issues and denied nasal symptoms. An outside magnetic resonance imaging scan of her brain was obtained that showed a left ethmoid mass measuring 2.4 × 2.3 × 2.5 cm extending into the left frontal sinus. A computed tomography sinus was then done in the office that revealed an expansile mass destroying the left lamina papyracea and fiberoptic laryngoscopy demonstrated a mass in the posterior nasopharynx adjacent to the left orbit and posterior to the left middle turbinate (Figure 1).

**Figure 2:**
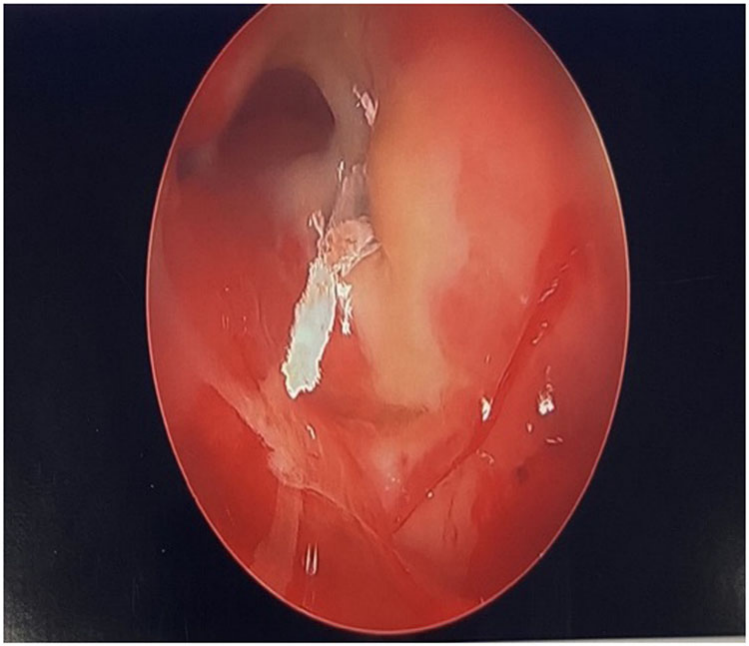
Fiberoptic photo. It displays a “knife” like object penetrating the left orbital tissue. The open frontal sinus can be seen above.

She was taken emergently to the operating room for functional endoscopic sinus surgery (ESS) under COVID restrictions and the frontal ethmoid mucocele was completely decompressed. A culture of the left frontal sinus was taken and did not show any fungal organisms. The patient then performed daily nasal saline irrigations and presented to postoperative appointments with mild orbital pain that decreased in intensity with each subsequent visit. Monthly nasal endoscopies consistently revealed an open, wide area at the site of the decompressed mucocele for three consecutive months. Three weeks later, she called the surgeon (N.L.S.) one Saturday night complaining of sudden onset, sharp pain that felt like she had a “knife in her left eye.” She was treated conservatively over the weekend with nasal irrigations and a nasal corticosteroid spray. A video endoscopy in the office revealed a sharp object penetrating the left orbital tissue (Figure 2). Attempted removal under video endoscopy failed and was painful as the spicule was impacted into the periorbital tissue that was now unprotected because the bony lamina papyracea had been resorbed by pressure from the mucocele. The object was finally irrigated under general anesthesia in the operating room. Spicule fragments were cultured and sent to pathology. Both reports were consistent with *Aspergillus fumigatus*. The patient's “knife‐like” pain was relieved after the procedure, but orbital pressure persisted until the fungal infection was brought under control.

## Discussion

Mucoceles most commonly occur in the frontal sinus (65%)^1^ and can be classified as primary or secondary depending on their origin site. Primary mucoceles usually begin as a cyst formed by a goblet cell gland and grow with the expansion of the sinus. Secondary mucoceles most commonly occur after sinus ostium obstruction, which blocks sinus drainage. This may result from chronic sinusitis, mucosal edema, surgical or nonsurgical trauma, or blockage caused by tumors.^2^

Patients with frontoethmoidal mucoceles typically present with orbit pain, diplopia, or proptosis^3^ and may present with nasal symptoms such as nasal obstruction.^1^ Mucocele treatment was dramatically altered after the development of ESS.^1^ External techniques, such as the osteoplastic frontal sinusotomy and craniotomy, were formerly used and required an incision to visualize the region of interest in the frontal and ethmoid sinuses. The goal was to fully eliminate mucoceles to prevent recurrence versus more modern‐day approaches that focus on drainage.^1^ External approaches are more invasive, resulting in higher morbidity, especially if bone erosion and orbital or intracranial extension of the mucocele are involved.^1^ cerebrospinal fluid leak, meningitis, and ocular cellulitis are all serious consequences of these surgical treatments.

In contrast, functional ESS avoids the potential complications of external approaches. It provides better clinical outcomes by preserving the frontal sinus mucosa, maintaining a patent frontal recess, and leaving no visible scars.^3^ There are three factors that might increase the risk of recurrence following ESS: surgery at a moment of acute infection, presence of multiple mucoceles, and significant extension outside the sinus wall.^4^ As in this case, the culture was positive for *Aspergillus fumigatus*. *Aspergillus* species are the most common cause of sinus fungal infections.^5^ It did not bear resemblance to a mycetoma, which usually appears under the skin. This patient probably formed the fungal spicule secondary to impaired orbital mucosa, exposed to the nose by mucocele destruction of the left lamina papyracea. The “mycelium sword” a phrase coined by the author as a metaphor for “Excalibur Sword” was treated postoperatively with antifungal irrigations consisting of itraconazole, which resolved the infection. Routine nasal saline irrigations may be considered postorbital mucocele depression until healing is complete.

## Author Contributions

**Nicholas L. Schenck**, case report; **Sarah A. Ustrell**, editorial oversight; **Zhiheng Chen**, references, editorial oversight.

## Disclosures

### Competing interests

None.

### Funding source

Cedars Sinai Medical Center Los Angeles.
